# Notes, Queries and Formulæ

**Published:** 1878-11-20

**Authors:** 


### *Practical Notes and Formulae.*

### BROMIDE POTASSIUM IN CHRONIC CHILLS.

Dr. L. T. S. of S. C. writes:

Case 1st. Mrs. P. Aged 65 has had third day chills for three years; commenced giving bromide, the morning after the chill gave 15 grains 3 times a day, has had no more chills for the last three years.

Case 2. Child of Mrs N. 5 years old has had third day chills for three years, gave bromide, 5 grain doses 3 times a day—has had no more chills.

The bromide is kept up for several months 3 times a day for 8 or 10 days, then left off for as many days.

I have been using the bromide as above for the last 6 or 7 years with uniform success. As a preventative—R. L. S. had chills every summer for several years—gave the bromide commencing in the spring; gave it all summer and fall at intervals as above; he has had no chills since, being now five or six years. I have seen no unpleasant effect whatever rom the use of bromide of potassium.

### VASELINE IN PHARMACY.

Having early last spring been engaged in preparing a few samples of cerates and ointments, with the above mentioned base, for the Paris Exposition, I made a ew different kinds, but rather as types indicative of the wide field which it covers. Vaseline is useful both on account of its inalterability when exposed to any ordinary atmosphere and its property of enveloping alterable bodies so as to render them stable. It is also a good solvent, and its freedom from granular structure renders the introduction of powders not perfectly impalpable visible, thereby insuring the physician against an oversight to which the pharmacist is liable with a granula ointment.

| | |
|---|---|
| Vaseline | 16 ounces. |
| White wax | 8 " |

Melt with a gentle heat. This cerate keeps well, is of medium consistence, and can be used all the year round, not being too hard for cold or too soft for warm weather.

| | |
|---|---|
| Vaseline | 16 ounces |
| Yellow wax | 4 " |
| Resin | 10 " |

Melt as resin cerate, U. S. P. This offers no advantage over our lard cerate, and requires constant stirring on cooling, as the resin tends to separate readily.—*Drug Cir.*

### ALTERATION OF CALOMEL.

Mr. Jolly has determined the amount of corrosive sublimate formed from 1 gram of calomel, after digesting it for six hours, at a temperature of 40 deg. C. (104 deg. F.), in 100 grams of distilled water to which the following additions had been made:

| | | |
|---|---|---|
| o·2 grm. muriatic acid | gave | ·oo3 HgCl2 |
| o· " sodium chloride | " | ·oo1 " |
| 2·o " citric acid | " | ·oo1 " |
| o·5 grm. soda | gave | ·oo6 HgCl2 |
| 1·o " sodium carb. | " | ·oo4 " |
| 1·o " magnesia | " | ·oo3 " |

Calcined magnesia and calomel, each 1 gram, were mixed; on washing the powder, after 24 houts, with distilled water, ·001 gram corrosive sublimate was obtained. Lime has the same effect; but, after digesting mixtures of calomel with carbonate of calcium or of magnesium and distilled water, no charge was observed after six hours. Troches of calomel made with pure sugar, free from lime, did not contain a trace of corrosive sublimate after having been kept for several months.—*L' Union Phar., Gaz. Medicale.*

*Asparagus root* is laxative, and acts also upon the kidneys.

---

*American Holly* (Hex opaca) is tonic, laxative and expectorant.

---

*Triostium perfoliatum,* is nervine and autispasmodic.

---

*Tinc. aconite.* The tincture of the radix, or root, is much more powerful than that of the leaves, a fact which should be borne in mind in using this powerful agent. It should also be remembered that when largely diluted with water it is more promptly absorbed, and more efficient in action.

---

*Deordorized tinc. opii.*, is perhaps the best form in which to administer this drug. It is less apt to disagree with the patient than the ordinary tincture.

---

*The muriate of Morphine* is used in England in preferance to the sulphate and acetate. It is not as likely to give rise to nausea and other unpleasant symptoms so common in the use of the sulphate. It is better,and we believe safer, for hypodermic use than the other forms mentioned.

---

*Virburum opulus,* or cramp bark, is a valuable austispasmodic—useful in asthma, spasms, cramps, etc. It is said to be the best and safest remedy for the relief of after pains :—Dose, one to two teaspoonful of the f. ext., repeated in an hour if necessary.

---

*Dog Fennel.*—One of the chamomile species, growing in the back lanes of our towns throughout the South, possesses the stomachic and tonic properties of the chamomile of the shops. In addition, the leaves of the plant contain a principal almost identical with cantharadine ; when bruised and applied to the skin, they produce prompt and active a epispastic effect.

---

*Nervine.*—The following is admirable to allay nervousness or morbid vigilance—specially useful to promote sleep in hysterical and irritable subjects, and is better for the baby than paregoric, or the various soothing syrups in use :

R Fluid ext. valerian

F. ext. skullcap a a.......... ½ oz.

F. ext. Catnip.......... 6 drs.

Dose for an adult, one to three drachms ; for a babe, 20 to 60 drops.

---

*Populus Tremuloides,* or American aspen is antispasmodic, tonic and alternative; useful as a febrifuge, in debility, as an anti-dyspeptic and in chronic diarrhœa. Dose of the f. ext. from a half to one drachm.

---

*Eczema Capitis.*

R. Plumbi acetatis.......... gr. x.
Zinci Oxidi..........
Hydrarg subcholoride..........
Ungt. hydarg nitratis a a.......... gr.
Adepis recentis.......... oz.
Olei palmae purificati.......... oz.j.

M. An anointment much relied on at the Skin Hospital—Blackfriars Road in the treatment of eczema capitis, and other obstinate eruptive diseases.

---

Bronchial Catarrh.—For a bad cold, particularly, for that forms of cartarrh attended by dry cough and constriction in the chest, or in mild form of asthma, the following remedy will be found efficient.

R. Pulo Doveri } .......... aa gr. v.

" Assafœtida } v.

Sulph. Quinine.......... gr. iij.

Take in one dose at bed time.

---

Carbolic Acid for Ague.—Dr. Stern, of Paris has found remarkable success in treating intermittent fever with the following formula.

R carbolic acid.......... gtt. vj.
Distil Water.......... oz. vj.

M. Dose, a tablespoonful three times a day.

---

For Rhumatism.

R. Acidi salicylici.......... dr. ii ;
Liq- ammon. acetatis......
Syr. limonis ....... aa f. oz ii ;

M. S.—Take two tablespoonfuls every wo hours.

